# Scaling up sanitation: Evidence from an RCT in Indonesia^☆^

**DOI:** 10.1016/j.jdeveco.2018.12.001

**Published:** 2019-05

**Authors:** Lisa Cameron, Susan Olivia, Manisha Shah

**Affiliations:** a University of Melbourne, Australia; b University of Waikato, New Zealand; c University of California, Los Angeles and NBER, USA

**Keywords:** Sanitation, Impact evaluation, Health, Scale up, Social capital

## Abstract

We investigate the impacts of a widely used sanitation intervention, Community-Led Total Sanitation, which was implemented at scale across rural areas of Indonesia with a randomized controlled trial to evaluate its effectiveness. The program resulted in modest increases in toilet construction, decreased community tolerance of open defecation and reduced roundworm infestations in children. However, there was no impact on anemia, height or weight. We find important heterogeneity along three dimensions: (1) poverty—poorer households are limited in their ability to improve sanitation; (2) implementer identity—scale up involves local governments taking over implementation from World Bank contractors yet no sanitation and health benefits accrue in villages with local government implementation; and (3) initial levels of social capital—villages with high initial social capital built toilets whereas the community-led approach was counterproductive in low social capital villages with fewer toilets being built.

## Highlights

• CLTS increased toilet construction and decreased tolerance of open defecation.
• The only significant health impact was a reduction in roundworms.
• No impacts on child haemoglobin counts, weight or height.
• Local government implementation was ineffective, unlike World Bank contractors.
• Level of community social capital was important to program success.

## 1 Introduction

It is estimated that about 1.1 billion people worldwide practice open defecation as a result of lack of access to sanitation facilities. Diseases caused by open defecation are preventable and disproportionately affect the poor. Millions of people contract fecal-borne diseases, most commonly diarrhea and intestinal worms, with an estimated 1.7 million people dying each year because of unsafe water, hygiene and sanitation practices (WHO/UNICEF, 2010). In Indonesia 110 million people lack access to proper sanitation and 63 million practice open defecation (WHO/UNICEF, 2012). Two of the four main causes of death for children under five in Indonesia (diarrhea and typhoid) are fecal-borne illnesses linked directly to inadequate water supply, sanitation, and hygiene issues (Ministry of Health, 2002). About 11 percent of Indonesian children have diarrhea in any two-week period and it has been estimated that more than 33,000 die each year from diarrhea (Curtis, 2004). By reducing normal food consumption and nutrient absorption, diarrheal diseases and intestinal worms are also a significant cause of malnutrition, leading to impaired physical growth (Guerrant et al., 1999), reduced resistance to infection (Baqui et al., 1993), and long-term gastrointestinal disorders (Schneider et al., 1978). Inadequate sanitation is associated not only with adverse health effects, but also with significant economic losses. Inadequate sanitation and poor hygiene in Indonesia is estimated to cost approximately US$6.3 billion, or more than 2.4 percent of the country's gross domestic product (GDP) (Napitupulu and Hutton, 2008).

Community-Led Total Sanitation (CLTS) is a program being widely implemented in more than 60 countries throughout Asia, Africa, Latin America, the Pacific and the Middle East to address the sanitation burden (Wells and Sijbesma, 2012). CLTS aims to create demand for sanitation by facilitating graphic, shame-inducing community discussions of the negative health consequences of existing sanitation practices, rather than through the more traditional approach of providing sanitation hardware or subsidies. The Water and Sanitation Program (WSP) of the World Bank is facilitating the implementation of CLTS widely. As part of a learning agenda to address the burdens associated with poor sanitation, the Bill and Melinda Gates Foundation funded randomized controlled trials (RCTs) of sanitation and hygiene interventions in seven locations around the world.^1^ This paper presents the results of the Indonesian evaluation. We report the impact of CLTS on outcomes of interest along the causal chain as improvements in sanitation have the potential to lead to a decrease in parasitic infestations, a decrease in anemia, and an increase in weight and height for young children. We rely on objective measures of impact—physical inspection of sanitation facilities, blood and fecal samples, and physical anthropometric measures. The evaluation results show that CLTS modestly increased the rate of toilet construction, decreased community tolerance of open defecation, and reduced the prevalence of roundworm infestation. There is no discernible impact on the other health measures and when we generate an overall health index including roundworm infestation, anemia, height and weight, there is no significant treatment effect. Allowing for heterogeneous treatment effects shows that the program is less effective among poorer households.

In addition to poverty status, we examine two other sources of heterogeneity in program impact. An important component of the intervention is that it sought to create a large-scale sustainable program across the country.^2^ WSP contracted resource agencies to implement the program in a set number of villages. The resource agencies were also contracted to train local government staff to implement the program themselves (partly through the observation of one or more of the resource agencies' implementations). During the phase of the project we evaluate, the resource agencies and local governments were implementing the program simultaneously. Approximately half of our treatment villages were treated by the resource agencies (RA) and the other half by local government (LG). This allows us to examine how program impact varies with the identity of the implementer and hence evaluate the scale-up process. As villages were not randomly allocated to implementing teams, the estimates examining this form of heterogeneity rely on the assumption that there are no unobserved differences between RA and LG villages which could be causing a differential impact. Discussions with WSP suggest there was no systematic process of assignment and tests of household and village baseline characteristics by implementer status show no significant differences. We find that while statistically significant program impacts are observed in communities where the program was implemented by a resource agency, local government implementation produced no discernible benefits.^3^

Second, we examine the role of social capital. As its name suggests, CLTS is a participatory development project in which facilitators are sent to villages to initiate a community analysis of existing sanitation practices and a discussion of the negative health consequences of such practices. The community actively participates in the facilitated meeting and is then left to forge its own plan to improve village sanitation with only limited follow-up support and monitoring from the program. Given CLTS' emphasis on community involvement, one might expect that it would function best in communities with high pre-existing levels of social capital. We define social capital as the norms and networks that enable collective action.^4^ Since social capital is not randomly allocated to villages, estimating these heterogenous treatment effects assumes that there are no unobserved variables which differ between low and high social capital communities that could be driving the results. While we cannot rule out that this might be the case, the stability of the estimates when a wide array of control variables are included suggests it is unlikely that the estimates are biased due to omitted variables (Altonji et al., 2005).

We find that baseline social capital is an important determinant of program effectiveness. High levels of community participation at baseline are strongly associated with increased toilet construction in treatment communities. However, in communities with low levels of social capital at baseline, significantly fewer toilets are constructed. We present results consistent with villages with higher initial community participation being more able to align community members' objectives with those of the program through the use of social sanctions. Our results thus constitute a caution about using participatory approaches in low social capital settings. At the very least they suggest that more intense involvement of project facilitators and general project support may be warranted in locations with demonstrably low social capital.

The paper proceeds as follows. Section 2 provides details of the intervention, the experimental design and data collection. Sections 3, 4 explain the estimation strategy and present the main impact evaluation results. Section 5 examines heterogeneous treatment effects. Section 6 concludes.

## 2 Intervention and study design

CLTS was initially developed in Bangladesh in 1999 by sanitation practitioner, Kamal Kar. It is now being widely implemented in more than 60 countries around the world (Wells and Sijbesma, 2012), having been adopted by many international NGOs (for example, Plan International, UNICEF, Care, World Vision) and the World Bank. Governments are increasingly taking the lead in scaling up CLTS with many having adopted CLTS as national policy. CLTS is viewed by many in the water and sanitation sector as the most promising approach to improving sanitation currently available.

The program is a community-led approach that focuses on creating demand for sanitation, in contrast to the traditional approach of supplying sanitation hardware (Sah and Negussie, 2009). CLTS facilitators are sent to villages to initiate a community analysis of existing sanitation practices and a discussion of the negative health consequences of such practices. The community actively participates in the facilitated meeting and is then left to forge its own plan to improve village sanitation with only limited follow-up support and monitoring from the program. These discussions, or “triggerings” are held in public places and are open to all. They involve a “walk of shame,” during which the facilitator helps people analyze how fecal contamination spreads from exposed excreta to their living environments and food and drinking water. A map of the village is drawn on the ground and villagers are asked to indicate where they live, where they defecate, and the routes they take there and back. This illustrates that everyone is ingesting small amounts of each other's feces which is intended to lead to individual and collective decisions to improve community health by becoming an open defecation free (ODF) community. ODF status is verified by local government agencies and community members.^5^

In contrast to other approaches that have been used widely in the past in Indonesia and elsewhere, no funding for infrastructure or subsidies of any kind is provided. CLTS founders believe that CLTS is less effective when subsidies are available (Kar and Pasteur, 2005). They argue that the existence of subsidies causes people to postpone investing in sanitation in the hope that they will receive a subsidy and that subsidies instill a culture of dependency rather than self-determination. The lack of subsidies also makes the program much less expensive and savings can be utilized to spread and scale up the{ }program.

### 2.1 Randomization design and data collection

In Indonesia CLTS was rolled out across all 29 rural districts in the province of East Java. East Java is Indonesia's second most populous province with approximately 38 million residents. Eight of the 29 rural districts in East Java were involved in the impact evaluation.^6^ In each district ten villages were randomly selected to participate in the impact evaluation as treatment villages and ten were randomly selected to act as control villages. The district offices were free to implement the program in other villages, other than the control villages. Most district offices implemented the program in 40–70 villages, with the program intending to reach a total of 1.4 million people across all rural districts in East Java (Cameron and Shah, 2010). Randomization was conducted at the village level, stratified by sub-district.^7^ There was only partial compliance with the randomization assignment. Of the 80 treatment villages, the endline survey data reports that 53 villages (66 percent) were triggered and 13.8 percent of the control villages were exposed to the program. Non-compliance was largely a result of district governments changing some of their target communities after the randomization plan had been agreed upon. Program administrative data collected as part of the CLTS program reports a higher percentage of treatment villages (83 percent) and a smaller percentage of control villages (4%) received the treatment. Below we estimate the average treatment effect across villages that were assigned to treatment, that is Intention-to-Treat (ITT) estimates.

Two waves of household data were collected. The baseline survey was conducted just prior to program implementation in August–September 2008. Within each village, approximately thirteen households were randomly selected to be surveyed. The endline data collection was conducted approximately 24 months later, between November 2010 and February 2011. The surveys collected a wide variety of information on the households including demographic information, a detailed sanitation module (including physical observations of household sanitation facilities which are used to verify household reports), and a child health module (including fecal samples to allow testing for parasitic infestations, blood tests for anemia, and anthropometric measurements). Fecal samples were collected by leaving a stool specimen container containing a preservative (formalin) with the child's carer and requesting that he/she deposit approximately 5 g of the child's feces in the container the next time the child defecates. The specimen was collected the next day. Preserved stool samples were sent to the public health office in Yogyakarta for analysis using the Kato-Katz technique (Katz et al., 1972). The fecal samples were tested for hookworm, roundworm, and whipworm. Prevalence of hookworm and whipworm was extremely low in the sample, with less that 1% testing positive for either of these types of worms, so we only use roundworm in the analysis. Hemoglobin was measured using HemoCues on a pinprick blood sample collected by trained field staff. Height and weight were measured using the standardized protocols developed by the Demographic and Health Surveys, see ICF International (2012).

To enable an examination of impacts on child health, households with children under the age of two were prioritized, with all surveyed households required to have at least one child under the age of five at baseline. Community level demographic data and information on infrastructure were also collected. In addition, a social capital module was conducted. Budgetary considerations restricted the social capital module to a randomly chosen six of these eight districts.^8^ Program administrative data identifies the implementing agency in each village.

Our total sample consists of approximately 2000 households spread across 160 rural villages in eight districts, with data on social capital available for approximately 1600 households spread across 120 villages in six of these districts.

## 3 Empirical strategy

Our empirical approach is to present ITT estimates of program impact on the outcomes of interest. This is done by estimating equation (1) below:

(1) $$Y_{ij}=\alpha +\beta_{1}T_{j}+\gamma X_{ij}+\delta_{K}+\varepsilon_{ij}$$

where *Y*_*ij*_ is the outcome measure for household *i* in village *j*; *T*_*j*_ is the treatment dummy, which equals 1 for households in the treatment group, and 0 otherwise; *δ*_*K*_ is a set of sub-district (*kecamatan*) dummy variables which are included because the randomization was stratified at this level. The sub-district effects also control for any differences in implementation across the eight districts. In some specifications, we also include a vector of household and village characteristics (*X*_*ij*_) as additional right-hand side control variables. The household variables are household size, the household head's age and educational attainment, household composition, log per capita household income, eligibility for low income support and dwelling characteristics; and the village variables are village population, village land area, the percentage of the village which is Muslim, whether there is a paved road to the nearest city, average years of education of household heads, whether a river flows through the village, and the percentage of households in the village who open defecated at baseline. *ε*_*ij*_ is the error term, and all specifications cluster the standard errors at the village level.

The causal average treatment effect is given by *β*_1_ if the randomization was effective. Table 1 uses the 2008 baseline survey data to compare characteristics of treatment and control groups. It shows that the means of a range of variables are similar in magnitude for the two groups and we cannot reject that they are equal for most of the variables. For the key outcome variables (sanitation, child health outcomes, attitudes toward open defecation), balance is achieved. The demographic and socio-economic characteristics are also similar across treatment and control groups. The baseline report provides tests of balance on a more extensive set of variables (Cameron and Shah, 2010).

**Table 1:** Summary statistics: Means and tests of balance.

Variable	All Villages			Treatment Villages		
	Treatment	Control	p–value	RA Treatment	LG Treatment	p–value
**Panel A. Baseline Household Characteristics:**						

Do not have own sanitation facility	0.52	0.48	0.44	0.52	0.52	0.95
Open defecate	0.40	0.40	0.93	0.40	0.39	0.89
Access to Unimproved Sanitation	0.11	0.12	0.76	0.12	0.10	0.66
Access to Improved Sanitation	0.49	0.48	0.74	0.48	0.51	0.69
Wash hands after defecation	0.99	0.99	0.20	0.99	0.99	0.80
Access to piped water	0.05	0.08	0.39	0.05	0.05	1.00
Intolerance of open defecation	33.1	32.9	0.70	33.6	32.6	0.19
Believes open defecation causes diarrhea	0.69	0.67	0.50	0.69	0.69	0.89
Knowledge of causes of diarrhea	4.73	4.73	0.94	4.81	4.66	0.39
Household head's age	40.4	40.4	0.99	40.2	40.5	0.68
Household head male	0.95	0.96	0.31	0.95	0.94	0.52
Household head's educational attainment:						
Elementary	0.52	0.48	0.27	0.53	0.51	0.68
Lower Secondary	0.20	0.21	0.46	0.19	0.21	0.68
Upper Secondary	0.18	0.21	0.28	0.19	0.18	0.74
Tertiary	0.04	0.04	0.78	0.04	0.05	0.76
Household size	4.92	4.82	0.29	5.00	4.83	0.20
No. children aged 0-5	1.15	1.12	0.07^∗^	1.15	1.15	0.85
No. children aged 6-10	0.33	0.31	0.45	0.32	0.34	0.56
No. children aged 11-17	0.39	0.40	0.65	0.39	0.39	0.91
Per capita household income	2.81	3.02	0.37	2.84	2.79	0.86
House has a dirt floor	0.22	0.23	0.91	0.25	0.20	0.33
House has a tiled floor	0.05	0.07	0.23	0.05	0.05	0.83
House has walls of brick or wood	0.88	0.89	0.86	0.87	0.90	0.30
Household uses wood as a cooking fuel	0.57	0.52	0.21	0.56	0.59	0.57
Cash transfer program (BLT) recipient	0.24	0.23	0.72	0.26	0.23	0.36
Max Observations:	922	936		460	462	

**Panel B. Baseline Child Characteristics:**						

Age in months	11.8	12.1	0.30	12.1	11.6	0.24
Male	0.50	0.52	0.44	0.49	0.51	0.48
Hemoglobin (g/l)	101.5	101.8	0.81	100.7	102.4	0.22
Weight (kgs)	8.2	8.3	0.61	8.2	8.3	0.49
Height (cms)	71.2	71.6	0.34	71.4	71.1	0.58
Max Observations:	946	940		471	475	

**Panel C. Baseline Village Characteristics:**						

Village population	1042	1300	0.14	824	1249	0.02^∗∗^
Paved road to nearest city	0.94	0.95	0.73	0.95	0.93	0.69
% of the village population that are muslim	97.6	95.4	0.19	97.4	97.9	0.67
River runs through village	0.72	0.71	0.86	0.72	0.73	0.89
% of households in village that open defecate	33.6	36.4	0.52	32.8	34.5	0.78
Average years of education of household heads	10.3	10.5	0.33	10.2	10.3	0.85
Village land area	42,816	34,930	0.70	42,377	43,234	0.98
Social capital index at BL	0.01	−0.02	0.61	−0.02	0.04	0.46
Max Observations:	80	80		39	41	

Note: These are summary statistics (means) using the baseline data. RA (LG) Treatment indicates villages which were assigned to implementation by a resource agency (local government). Information on roundworm prevalence and intolerance of open defecation is not available at baseline. The p-values are generated from tests of statistical difference between treatment and control communities.

When examining heterogeneity of impact, we include interactions of *T*_*j*_ with the relevant variables – poverty status of the household; whether the village was assigned to be treated by a resource agency (RA) or by the local government (LG); and baseline social capital. We discuss the issue of balance with respect to the relevant sub-samples in the analyses below.

## 4 Empirical results

Table 2 reports the ITT estimates for the main outcomes of interest—toilet construction, attitudes toward open defecation, knowledge of the causes of diarrhea and child health outcomes. Control variables are included in these specifications. Table A1 in the appendix reports results when control variables are not included. The results are similar.

**Table 2:** Does CLTS treatment improve sanitation and child health outcomes?

*Dependent Variables:*	Toilet Construction		Intolerance of Open Defecation		Diarrhea Knowledge	Roundworm (eggs/g)		Hemoglobin (g/l)	Weight z-score	Height z-score	Health Index
	(1)	(2)	(3)	(4)	(5)	(6)	(7)	(8)	(9)	(10)	(11)
Treatment	0.024 (0.014)^∗^		0.409 (0.233)^∗^		0.007 (0.058)	−72.81 (39.55)^∗^		−0.18 (0.51)	0.03 (.03)	−0.01 (.03)	0.02 (.02)
RA Treatment^∗^ Poor		−0.027 (0.034)		0.644 (0.56)			−71.4 (110.5)				
RA Treatment^∗^ Non-poor		0.072 (0.028)^∗∗∗^		1.035 (0.312)^∗∗∗^			−176.4 (67.2)^∗∗∗^				
LG Treatment^∗^ Poor		−0.028 (0.036)		0.069 (0.553)			35.9 (83.8)				
LG Treatment^∗^ Non-poor		0.014 (0.016)		−0.128 (0.316)			4.9 (48.4)				
Poor		0.009 (0.022)		−1.031 (0.359)^∗∗∗^			−69.9 (65.9)				
Mean DV (Treat = 0)	0.125	0.125	32.9	32.9	4.85	156.7	159.2	111.0	−1.39	−1.65	0.001
Controls:	Yes	Yes	Yes	Yes	Yes	Yes	Yes	Yes	Yes	Yes	Yes
Tests of Equality (p-values):											
RA^∗^ Non-Poor = RA^∗^ Poor		0.02		0.49			0.37				
LG^∗^ Non-Poor = LG^∗^ Poor		0.31		0.74			0.37				
RA^∗^ Non-Poor = LG^∗^ Non-Poor		0.06		0.004			0.01				
RA^∗^ Poor = LG^∗^ Poor		0.98		0.40			0.31				
RA^∗^ Poor = RA^∗^ Non-poor =		0.09		0.04		0.68	0.09				
LG^∗^ Poor = LG^∗^ Non-poor											
Observations	1858	1858	1858	1858	1858	1780	1742	1443	1886	1872	2043

Notes: We report results from OLS regressions (equations (1), (2))). The dependent variables are: *Toilet Construction* which equals 1 if the household built a toilet since baseline and 0 otherwise; *Intolerance of Open Defecation* which is the sum of responses to 9 questions about attitudes toward open defecation (45 is the maximum score possible and is the highest level of intolerance while 9 is the minimum score possible and reflects total acceptance of open defecation); *Diarrhea Knowledge* which is a score out of 6 based on six questions about possible causes of diarrhea (a score of 6 indicates that the respondent got all of the questions correct); roundworm prevalence (eggs/g); hemoglobin (g/l); weight and height z-scores of children 0–5; and an index of roundworm, hemoglobin, weight z-scores, and height z-scores. RA (LG) treatment indicates villages are assigned to implementation by a resource agency (local government). Standard errors are clustered at the village level and are reported in parentheses. All specifications include sub-district fixed effects and household control variables (household size, the household head's age and educational attainment, household composition, log of per capita household income, eligibility for low income support and dwelling characteristics) and village control variables (the village population, village land area, the percentage of the village which is Muslim, whether there is a paved road to the nearest city, average years of education of household heads, whether a river flows through the village, and the percentage of households in the village who open defecated at baseline). Columns 6–11 also control for the sex of the child, and dummy variables for age in months of the child. ^∗∗∗^indicates significance at 1% level, ^∗∗^ at 5% level, ^∗^ at 10% level.

We first examine whether CLTS treatment was successful in stimulating demand for sanitation (column 1). Households report whether they built a toilet between baseline and endline. This report is verified at the end of the interview by an inspection of the household's sanitation facilities. Table 2 shows that treatment increases toilet construction by 2.4 percentage points. This constitutes a 19 percent increase in toilet construction relative to control communities.

CLTS is hypothesized to stimulate demand for sanitation by inducing shame associated with open defecation. It also imparts information on the negative health consequences of poor sanitation. Column 3 reports the results of whether treatment impacts a measure reflecting the degree to which the respondent agrees (disagrees) with negative (positive) views of open defecation. There is a small decrease in the community's tolerance of open defecation in treatment communities relative to control communities. The program does not affect knowledge of the causes of diarrhea (unclean water, not washing hands, open defecation, etc.) which may be due to knowledge being quite high already, with the mean score in control communities being 4.9 out of 6 (column 5).^9^

The ultimate aim of CLTS is to improve sanitation so as to improve community health, particularly child health. We examine the health impacts along the causal chain from roundworm infestation, to hemoglobin blood concentrations (with low hemoglobin indicating anemia) to weight and height z-scores (columns 6–11). While columns 1–5 are estimated at the household level, the health regressions are estimated at the child level. The sample of children is those aged 0 to 5 at endline.

Column 6 of Table 2 shows that treatment is associated with an approximately 46% decrease in roundworm infestation. This is a large decrease, which is surprising given the relatively modest increase in access to sanitation. Such a large decrease in roundworms would have the potential to have significant impacts on nutritional intake and so might be expected to be reflected in anemia, weight or height gains. However, we do not find any significant treatment effects on hemoglobin concentrations, weight or height z-scores.

In column 11 we generate an overall health index. Following Kling et al. (2007) and Casey et al. (2012), we construct the index by orienting all variables so that the positive direction indicates a better outcome; demeaning all re-oriented outcomes and dividing each variable by the control group standard deviation; and calculating the average of these variables. While the coefficient on treatment is positively signed, it is not statistically significant (column 11).

## 5 Heterogeneous treatment effects

### 5.1 Scale up

CLTS forms part of the Indonesian government's national strategy to improve environmental and health outcomes in rural areas. Ensuring sustainability of the project by embedding implementation in district governments was the key element of the scale up strategy. WSP followed the World Health Organization's documented steps for developing a successful scale up strategy which were formed on the basis of evidence gathered over years of experience in scaling up public health interventions (WHO, 2010).^10^

The scale up model used was the widely-employed “Training of Trainers” (Binswanger and Nguyen, 2004). WSP trained staff at resource agencies (RA) which had successfully bid for the work and then these resource agencies trained local government (LG) officials in CLTS with the local government then taking over and scaling up the program to all villages (Rosenzweig and Kopitopoulos, 2010). A portion of this training was done by demonstration or “learning-by-doing”, as LG officials observed RAs triggering some villages. This process took place at the time of the RCT. Amongst the treatment villages in our sample, we have 39 villages triggered by the RAs and 41 villages triggered by the LGs.

There are a number of reasons why program impacts may differ when conducted at scale:

1. *Demographic context.* Different characteristics of target populations when at scale.
2. *Design effects.* Differences in program design that are necessary to bring the program to scale.
3. *Scale effects.* General equilibrium effects associated with the scale of the project, including spillover effects.
4. *Implementation agent effects.* The identity of the implementing agency may alter incentives.

In our context, many of these effects are not present. For example, as the program was implemented simultaneously by both RAs and LGs, any general equilibrium effects will be common to both treatment types. General equilibrium effects could include spillovers resulting from widespread increased demand for sanitation (e.g. latrine price changes and spillovers in information about the benefits of sanitation from treatment villages to control villages). In addition, the geographic and demographic context did not differ systematically and the project design is identical. Hence, it is a situation where there is a reasonable likelihood of successful scale up being achieved. The only potential difference between RA and LG implementation is in the *implementation agent* effect. That is the implementing actors and their associated administrative constraints differed.^11^

Villages were not randomized into LG vs RA status, though discussions with WSP suggest there was nothing systematic about how these decisions were made. If the characteristics of villages differ with implementing agency then we could falsely ascribe differences in program effectiveness to LG versus RA triggering. Tests of whether villages that were assigned to be triggered by local governments are otherwise similar to the villages that were assigned to be triggered by resource agencies are presented in columns 4–6 of Table 1. Table 1 shows the villages are remarkably similar. There are no observable differences in the demographic and socio-economic composition of the villages. There are also no significant differences in access to sanitation or open defecation rates at baseline. This is important because if local governments are cherry-picking villages so as to work with communities that are most likely to become open defecation free then we would expect to see differences in baseline sanitation.

We also examine differences at the village level which might influence the population's interest in sanitation (panel C, Table 1). There are no differences in most of the village characteristics, including the accessibility of the villages (having a paved road to the nearest town and the distance to the city), levels of social capital, and whether a river runs through the village. Defecating in rivers is common practice in Indonesia and CLTS field workers report that motivating households to build toilets in villages that are on a river is more difficult (Mukherjee, 2011). There is also no difference in the percentage of households in the village that open defecate at baseline. The only difference we observe is that the RA-assigned villages have a significantly smaller population than LG-assigned villages. We control for village population in the specifications below. Population is not a significant determinant of the probability of building a toilet nor of the key health outcomes.

#### 5.1.1 Poverty status

As discussed earlier, program impact may also vary with the poverty status of the household, as CLTS does not provide financial assistance in any form to poorer households, the ability of poorer households to participate in the program by constructing a toilet may be limited. Toilet construction requires a significant outlay of capital. In the endline survey, cost is the most frequently reported obstacle to building a toilet, reported by 47% of households. Further, less than 5% of poor households report having sufficient savings to cover the cost of building a latrine (estimated by WSP to be USD 46).^12^ Credit is rarely used as a financing mechanism, likely due to lack of availability and/or high interest rates. Only 2.5% of households who built a toilet report borrowing to do so.

We examine the heterogeneity of program impact by poverty status and implementer identity simultaneously. We generate an indicator to identify poor households with a household being deemed poor if they are in the bottom quartile of the distribution of non-land assets.^13^ We include this variable in the regressions interacted with treatment status and implementer identity.

#### 5.1.2 Estimation strategy

Equation (2) is the estimating equation that allows for treatment effects to differ both by poverty status and the identity of the implementer. We note that we only test for heterogeneity among the dependent variables with a statistically significant average treatment effect (toilet construction, intolerance of open defecation, and roundworm). The outcome measures are regressed on a treatment dummy interacted with both implementing agency and poverty status. $T_{ij}^{RA*Poor}$ equals one if household *i* in village *j* is poor and village *j* was assigned to be triggered by an RA and zero otherwise; $T_{ij}^{RA*Nonpoor}$ equals one if household *i* in village *j* is not poor and village *j* was assigned to be triggered by an RA and zero otherwise. $T_{ij}^{LG*Poor}$ and $T_{ij}^{LG*Nonpoor}$ are defined analogously when village *j* is assigned to local government implementation. The estimating equation is:

(2) $$\begin{array}{cc} Y_{ij} & =\alpha +\beta_{1}T_{j}^{RA*Poor}+\beta_{2}T_{j}^{RA*Nonpoor}+\beta_{3}T_{j}^{LG*Poor}+\beta_{4}T_{j}^{LG*Nonpoor}\\ & \;+\gamma X_{ij}+\delta_{K}+\varepsilon_{ij} \end{array}$$

The coefficients of interest are *β*_1_, *β*_2_, *β*_3_, and *β*_4_. Comparisons of these coefficients reveals the differential impact of implementing agency for poor and less poor households. All other variables are as defined previously.

#### 5.1.3 Results

Column 2 in Table 2 presents the results for toilet construction. Toilet construction increases significantly only among less poor households in treatment villages where the program was implemented by a resource agency. Non-poor households in these villages increased their toilet construction by 7.2 percentage points relative to non-poor households in control villages. The impact on toilet construction by poorer households in these treatment villages is not significant statistically (with a negative point estimate) and there are no significant impacts for households, whether poor or non-poor, in treatment communities where implementation was by local government. The difference in treatment impact between poor and non-poor households in RA treatment villages is statistically significant (p = 0.02), as is the difference between the impact on non-poor households in RA and LG villages (p = 0.06). We can also reject that all of the coefficients on the treatment variables are equal (p = 0.09).

These results call into question CLTS's strategy of not providing subsidies for toilet construction since poorer households are no more likely to build toilets in treatment communities than in control communities, and the cost of construction is reported as a main obstacle to improving sanitation. In fact, recent empirical evidence suggests a crucial role for subsidies (Dupas, 2014). Patil et al. (2014) and Pattanayak et al. (2009) evaluate India's Total Sanitation Campaign which uses a CLTS approach with subsidies. They find significant increases in access to improved sanitation but no robust health impacts. Hammer and Spears (2016) also study the Total Sanitation Campaign in the Indian state of Maharashtra and find the program has a large positive impact on children's heights. Guiteras, Levinsohn and Mobarak (2015) show that in Bangladesh, subsidies to the poor increase toilet ownership both among subsidized households and their unsubsidized neighbors, which suggests that investment decisions are interlinked across neighbors.

Column 4 presents results of the same specification but with the score for intolerance of open defecation as the dependent variable. Interestingly, the results for attitudinal change show a corresponding decrease in tolerance of open defecation among the non-poor in communities where a resource agency is the implementing agency, suggesting that the resource agencies are more effective at generating attitudinal change and that the program has difficulty affecting the attitudes of households who are least able to afford sanitation.

We also examine whether these differential improvements in sanitation infrastructure by triggerer identity and poverty status are apparent in the roundworm finding. Column 7, Table 2 shows that the reductions in roundworm are concentrated among less poor households in RA villages. In contrast, this coefficient for LG villages is not statistically significant (and close to zero). The difference between RA and LG villages is significant at the 1% level.^14^

#### 5.1.4 How does RA implementation differ?

Table 3 compares facets of program implementation across treatment villages triggered by RA or LG. We investigate whether the way information was disseminated to the community (panel A); the extent of program engagement with village staff (panel B); the intensity of implementation and the use of rewards or competitions (panel C), and the extent of community participation (panel D) were different between RA and LG villages. We first present an overall measure for each panel and then include results for the individual variables that contribute to these measures in the subsequent columns. The summary measure for each overall indicator is defined in the table notes in Table 3.

**Table 3:** Did RA implementation differ from LG implementation?

**Panel A. Information Dissemination**							
	*Aggregate Indicator*	*TV*	*Radio*	*Print*	*Video*	*Shop Sign*	*Village Notice*
	(1)	(2)	(3)	(4)	(5)	(6)	(7)
Treated by RA	0.05 (0.06)	−0.002 (0.05)	0.04 (0.03)	0.04 (0.05)	0.02 (0.01)	0.03 (0.03)	0.04 (0.04)
Mean DV	0.48	0.40	0.14	0.17	0.02	0.07	0.12
Observations	635	635	635	635	635	635	635

**Panel B. Local Engagement**							
	*Aggregate Indicator*	*Health Officer*	*Midwife*	*Health Post Volunteers*	*Village Office*		
	(8)	(9)	(10)	(11)	(12)		
Treated by RA	0.15 (0.06)^∗∗^	0.09 (0.06)	0.06 (0.06)	0.11 (0.06)^∗^	0.11 (0.04)^∗∗∗^		
Mean DV	0.49	0.33	0.31	0.34	0.14		
Observations	635	635	635	635	635		

**Panel C. Implementation Intensity**							
	*Aggregate Indicator*	*No. of Facilitators*	*Facilitator Charisma*	*No. of Visits*	*Rewards or Competition*		
	(13)	(14)	(15)	(16)	(17)		
Treated by RA	0.45 (0.23)^∗^	1.3 (0.77)^∗^	0.16 (0.15)	0.42 (0.22)^∗∗^	−0.04 (0.11)		
Mean DV	−0.07	3.19	2.8	0.91	0.10		
Observations	635	635	635	635	635		

**Panel D. Community Participation**							
	*Aggregate Indicator*	*Heard about Program*	*Know about Triggering*	*Attended Triggering*			
	(18)	(19)	(20)	(21)			
Treated by RA	0.34 (0.13)^∗∗^	0.13 (0.06)^∗∗^	0.12 (0.05)^∗∗^	0.08 (0.05)			
Mean DV	0.90	0.66	0.16	0.10			
Observations	635	635	635	635			

Notes: The sample is restricted to observations in villages which were treated. We report the coefficient on the indicator that the village was treated by a resource agency (RA). *Information Dissemination Aggregate Indicator* equals 1 if any of TV, radio, print media, video, notices in shop windows or village notice boards were used to disseminate information about the program, 0 otherwise. The other dependent variables in Panel A equal 1 if the respondent heard about the sanitation program from the specified source, and 0 otherwise. In Panel B, *Local Engagement Aggregate Indicator* equals 1 if the program engaged with any of village health officers, midwives, health post volunteers or village officials, 0 otherwise. The dependent variables in columns 9–12 equal 1 if the program engaged with the specified officers, 0 otherwise. *Implementation Intensity Aggregate Indicator* is an unweighted standardized index of the variables in columns 14–17. *No. of Facilitators* is the village average of respondents reports of how many facilitators were at the triggering; *Facilitator Charisma* is the village average of respondent rankings from 1 to 4 of how charismatic/persuasive the facilitators was; *Number of visits* is the village average of respondent reports of how many visits the facilitators made to the village, and *Rewards or Competition* equals 1 if one or more respondents in the village reported that the program involved rewards for villages becoming open defecation free and/or competitions between villages with regard to decreasing open defecation. *Community Participation Aggregate Indicator* is the sum of the dependent variables in columns 19–21 which are indicators of whether the household had heard of CLTS, knew about the triggering and had attended the triggering. Standard errors are clustered at the village level. ^∗∗∗^indicates significance at 1% level, ^∗∗^ at 5% level, ^∗^ at 10% level.

There is no significant difference in the way the RAs and LGs disseminated information about the project (via TV, radio, print media, video, notices in shop windows or on village notice boards). The RAs are however more likely to engage with village staff, in particular, with the village office and with village health post volunteers. The intensity of implementation is greater in RA villages (driven by a greater number of facilitators visiting the communities, and facilitators making more visits). Most villages received only one visit from the team, some villages received two visits and a small number received three. RA facilitators made 0.42 more visits to villages than LG teams (significant at the 5% level). RA implementation also results in significantly greater community participation. Respondents in RA-triggered treatment villages are 13 percentage points more likely to have heard about the program and 12 percentage points more likely to have known about the triggering event.

In the field one hears a lot about the importance of the “quality” of the facilitator. In order to test whether the RA facilitators are “better” than the LG facilitators, we collected information from respondents on their perceptions of how charismatic/persuasive the facilitators were. We find no significant difference in the average reported persuasiveness of the facilitators (column 15). An examination of various program reports reveals that there was general satisfaction of WSP staff with the quality of the RA training of facilitators (Rosenzweig and Kopitopoulos, 2010).

### 5.2 Role of social capital

Given the community-led, participatory nature of CLTS it seems likely that initial levels of social capital in treatment villages would impact on program effectiveness. A higher level of social capital is thought to facilitate collective action by lowering the costs associated with such action (Casey et al., 2012); by reducing the problem of free riding; and facilitating the transmission of knowledge about the behavior of others and hence reducing the problems of opportunism (Collier, 1998). Communities with a greater degree of pre-existing community interaction are likely to be better prepared to cooperate and also have a greater store of community knowledge on which to draw when targeting the poor and prioritizing community needs. Conversely, communities with low stocks of social capital might struggle to work together and agree on community priorities.

High levels of social capital within a community may also result in households internalizing the social benefit of the provision of private goods, (Karlan, 2005). If a household builds a toilet and stops defecating in the village stream then other villagers benefit from this. If communities with higher levels of social capital internalize these social benefits more, they are likely to be more interested and willing to work together to improve the community's sanitation. Research on the relationship between initial levels of social capital and outcomes of participatory development programs is almost non-existent and where it does exist, data limitations mean that the direction of causality is not clearly identified.^15^ Reports from field workers suggest that the CLTS approach is particularly effective in settlements with a sense of community (Chambers, 2009).^16^

#### 5.2.1 Social capital empirical strategy and results

To examine the impact of social capital on the success or otherwise of the sanitation program we construct a village social capital index from data collected at baseline on participation in community groups and the extent of networks in the village. The index is constructed in the same way as we calculated the index of health outcomes above, following Kling et al. (2007). Table A2 in the appendix provides details of the household-level social capital variables collected at baseline that are used to construct the index. As we are interested in village level social capital, the index is calculated from village averages of each of these variables.

Table A2 shows that the social capital variables are balanced between treatment and control. There are no significant differences in any of the individual social capital variables, nor in the village social capital index. This is true across the entire sample and also within the sub–sample of households who do not have sanitation at baseline. We estimate the social capital regressions over households that had no access to sanitation at baseline so as to focus on households which can improve their sanitation through building a toilet in response to the program. In the previous sections when we were looking at both toilet construction and health outcomes, we examined estimates over the whole sample as health benefits may accrue to those who built a toilet as well as other households in the community. Table A3 additionally shows that access to sanitation, improved water sources and sanitation behavior (handwashing) is balanced at baseline in the sub-sample for which we have social capital data.

We allow for program impact to differ with the level of baseline social capital (and implementing agency and poverty status) by adding three additional regressors to equation (2). The additional variables are the index of social capital at baseline; the index of baseline social capital interacted with a treatment dummy; and the index of baseline social capital interacted with RA treatment.

The estimating equation is:

(3) $$\begin{array}{cc} Y_{ij} & =\alpha +\beta_{1}T_{j}^{RA*Poor}+\beta_{2}T_{j}^{RA*Nonpoor}+\beta_{3}T_{j}^{LG*Poor}\\ & \;+\beta_{4}T_{j}^{LG*Nonpoor}+\beta_{5}T_{j}*SC_{j\left[BL\right]}+\beta_{6}T_{j}^{RA}*SC_{j\left[BL\right]}\\ & \;+\beta_{7}SC_{j\left[BL\right]}+\gamma X_{ij}+\delta_{K}+\varepsilon_{ij} \end{array}$$

The new coefficients of interest are *β*_5_, *β*_6_, and *β*_7_. Everything else is as before.

The results from estimating equation (3) for toilet construction are reported in Table 4. We first show the average treatment effect for this smaller sub-sample. Column 1 shows that households in the social capital sample who did not have sanitation at baseline are 6 percentage points more likely to build a toilet than like households in control villages. Columns 2 and 3 include the interactions between treatment status, implementer identity, poverty status, and baseline village social capital (with and without controls). The level of social capital at baseline in treatment villages is strongly positively associated with the probability of toilet construction, particularly in RA treated villages. A one standard deviation increase in the baseline community participation index is associated with approximately an 11.3 percentage point (188%) increase in the probability that a household built a toilet (significant at the 5% level). This is a large increase and signifies substantial variation in program success dependent on the initial level of community participation. The program impacts are concentrated among the non-poor in RA villages with high levels of baseline social capital. A comparison of these results with and without controls establishes that the inclusion of controls does not substantially alter the estimated impacts.

**Table 4:** Does social capital impact toilet construction?

	(1)	(2)	(3)	(4)	(5)
Treatment	0.06 (.02)∗∗∗				
RA Treatment^∗^ Poor		0.07 (0.08)	0.10 (0.08)	−0.04 (0.15)	−0.003 (0.07)
RA Treatment^∗^ Non-poor		0.13 (0.04)∗∗∗	0.11 (0.04)∗∗	0.07 (0.05)	0.09 (0.05)∗
LG Treatment^∗^ Poor		−0.01 (0.06)	−0.11 (0.06)∗	0.01 (0.1)	0.007 (0.07)
LG Treatment^∗^ Non-poor		0.02 (0.03)	−0.02 (0.03)	−0.01 (0.04)	−0.008 (0.03)
Treatment^∗^ Village Social Capital BL		0.18 (0.08)∗∗	0.14 (0.08)∗		
RA Treatment^∗^ Village Social Capital BL		0.17 (0.15)	0.33 (0.16)∗∗		
Treatment^∗^ Quintile 1 of Village Social Capital BL				−0.14 (0.06)∗∗	−0.16 (0.04)∗∗∗
Treatment^∗^ Quintile 2 of Village Social Capital BL				−0.02 (0.08)	
Treatment^∗^ Quintile 3 of Village Social Capital BL				0.04 (0.06)	
Treatment^∗^ Quintile 4 of Village Social Capital BL				−0.04 (0.06)	
Treatment^∗^ Quintile 5 of Village Social Capital BL				−0.06 (0.06)	
RA Treatment^∗^ Quintile 1 of Village Social Capital BL				−0.07 (0.1)	
RA Treatment^∗^ Quintile 2 of Village Social Capital BL				0.14 (0.09)	
RA Treatment^∗^ Quintile 3 of Village Social Capital BL				−0.01 (0.08)	
RA Treatment^∗^ Quintile 4 of Village Social Capital BL				0.37 (0.11)∗∗∗	0.27 (0.09)∗∗∗
RA Treatment^∗^ Quintile 5 of Village Social Capital BL				0.28 (0.09)∗∗∗	0.18 (0.06)∗∗∗
Village Social Capital BL		0.03 (0.14)	−0.11 (0.13)	−0.35 (0.12)∗∗∗	−0.28 (0.11)∗∗∗
Poor		−0.04 (0.03)	−0.02 (0.03)	−0.03 (0.03)	−0.03 (0.03)
Controls	No	No	Yes	Yes	Yes
Mean DV (Treatment = 0)	0.06	0.06	0.06	0.06	0.06
Test Q1 = Q2 = Q3 = Q4 = Q5				< 0.001	
Test RA^∗^ Q1 = RA^∗^ Q2 = RA^∗^ Q3 = RA^∗^ Q4 = RA^∗^ Q5				< 0.001	
Observations	596	596	596	596	596

Notes:These are OLS regressions on the sample of households that did not have access to sanitation facilities at baseline for the social capital sample from equations (1), (3)). All specifications include sub-district fixed effects. Standard errors clustered at the village level and are reported in parentheses. ^∗∗∗^indicates significance at 1% level, ^∗∗^ at 5% level, ^∗^ at 10% level. RA (LG) Treatment indicates villages assigned to implementation by a resource agency (local government). Village Social Capital BL is the baseline village social capital index constructed from the variables in Table A2. Column 5 drops the interactions with quintile of the baseline village social capital which are not statistically significant in column 4. Columns 3–5 include the usual set of controls – household control variables (household size, the household head's age and educational attainment, household composition, log of per capita household income, eligibility for low income support and dwelling characteristics) and village control variables (the village population, village land area, the percentage of the village which is Muslim, whether there is a paved road to the nearest city, average years of education of household heads, whether a river flows through the village, and the percentage of households in the village who open defecated at baseline).

Columns 4 and 5 in Table 4 allow for non-linearities in the impact of baseline village social capital. We interact treatment with indicators of quintiles of the distribution of the baseline social capital index. Toilet construction is spurred by being in the top two quintiles of the social capital distribution in RA treatment villages. In villages with very low levels of community participation (lowest quintile) toilet construction is approximately 16 percentage points lower in treatment villages than in similar control villages. The linear model also predicts that fewer toilets are constructed in treatment communities than control communities when social capital is low. In the raw data, 11 percent of households constructed toilets in treatment communities in the lowest quintile of the social capital distribution compared to 20 percent in similar control communities.

#### 5.2.2 Mechanisms for the social capital result

We test three hypotheses to better understand what might be driving the social capital results. First, a more active community may mean a better informed community as members know each other better and exchange information when they meet. To examine this hypothesis we construct an index of information shared within the community at endline from variables reflecting whether the household reported that they knew about the triggering event; whether they learned about sanitation construction from other community members; and whether knowledge about the causes of diarrhea increased between baseline and endline.^17^ Whether villages with higher levels of baseline participation have greater information flows at endline is tested by regressing this index on baseline village social capital. Baseline social capital is not associated with significantly greater information flows (see Table 5, column 1).

**Table 5:** Role of baseline social capital in treatment communities.

Dependent Variable:	Information Index EL (1)	Sharing/Attendance Index EL (2)	Sanctions Index EL (3)
Village Social Capital Index BL	0.13 (0.13)	0.09 (0.15)	0.51 (0.18)∗∗∗
Control variables	Yes	Yes	Yes
Sub-district fixed effects:	Yes	Yes	Yes
Mean DV (treatment = 0)	0.01	0.00	0.00
Observations	721	721	721

Notes: These are OLS regressions for the entire sample of treatment households for which we have social capital data. Village Social Capital BL is the baseline village social capital index constructed from the variables in Table A2. All regressions include household control variables (household size, the household head's age and educational attainment, household composition, log of per capita household income, eligibility for low income support and dwelling characteristics) and village control variables (the village population, village land area, the percentage of the village which is Muslim, whether there is a paved road to the nearest city, average years of education of household heads, whether a river flows through the village, and the percentage of households in the village who open defecated at baseline). Standard errors clustered at the village level and are reported in parentheses. ^∗∗∗^indicates significance at 1% level, ^∗∗^ at 5% level, ^∗^ at 10% level.

Second, in more active communities, people may be more willing to be actively involved and share resources as a result of knowing each other better. In the CLTS context this may result in being more likely to attend the triggering event and more shared and public toilets being built. To test this hypothesis we construct an index from these two variables and examine its relationship with the baseline social capital index. Baseline social capital is not a significant determinant of active involvement and sharing of resources (see Table 5, column 2).^18^

The final mechanism we examine is whether social sanctions play a greater role in encouraging households to build toilets in communities with more social capital. If household members know their community better, they may be more concerned about what other community members think of them. We construct an index of sanctions from reports on a scale of 1 (strongly agree) to 5 (strongly disagree) on whether building a toilet reduces the likelihood of being a target of gossip; whether those who defecate in the open will not be accepted by the community; and whether the community imposes social sanctions on those who defecate in the open. Table 5 (column 3) shows that villages with higher levels of social capital at baseline are more likely to impose sanctions, consistent with these communities being more able to regulate behavior by the use of social opprobrium.

## 6 Conclusion and policy implications

CLTS modestly increased the rate of toilet construction and reduced community tolerance of open defecation. We find an associated decrease in roundworm infestations but no improvements in children's hemoglobin levels, weight or height. An index of child health also shows no significant overall improvement. Although the rate of toilet construction increased about four percentage points among less poor households, the poorest households did not build toilets. This highlights potentially important roles for the provision of finance to poor households and/or subsidies for the poor in conjunction with CLTS in producing open defecation free communities (and the possible concomitant health benefits).

The examination of the scale up process shows that CLTS had relatively large positive impacts in villages where the program was implemented by RAs. In contrast, with the identical program design, the same demographic composition of participating households, and common general equilibrium effects, implementation by local governments failed to produce any discernible positive impacts. Understanding what makes for successful scale up is of prime importance to the development sector. Currently there are very few studies that explicitly examine the scale up process through the lens of a rigorous quantitative evaluation and the studies that exist find either a lack of replicability at scale or that successful scale up is not straightforward and involves considerable learning from failure. Integration of quantitative evaluation, qualitative research, and high quality monitoring data is likely to improve researcher and program implementers' ability to understand the causes of success and failure, so as to increase the likelihood of successful scale up in the future.^19^

Finally, our results show that CLTS increased toilet construction in villages with sufficiently high pre-existing social capital in the form of community participation. In villages with low initial levels of social capital, however, the program was counterproductive—resulting in fewer toilets being built. We present evidence consistent with high social capital communities being better able to use social pressure to get community members to conform with program objectives. Our finding are thus cautionary with respect to using participatory development approaches in low social capital environments and at the very least suggest a need for greater investment in community-support for participatory development programs in areas with demonstrably low social capital.

**Table A1:** Does CLTS improve sanitation and child health outcomes? (No Controls).

Dependent Variables:	Toilet Construction		Intolerance of OD		Diarrhea Knowledge	Roundworm (eggs/g)		Hemoglobin (g/l)	Weight z-score	Height z-score	Health Index
	(1)	(2)	(3)	(4)	(5)	(6)	(7)	(8)	(9)	(10)	(11)
Treatment	0.03 (0.01)∗∗		0.28 (0.28)		−0.01 (0.05)	−58.9 (35.5)∗		0.07 (0.45)	0.02 (0.03)	−0.03 (0.03)	0.03 (0.02)
RA Treatment^∗^ Poor		−0.03 (0.03)		0.78 (0.64)			−39.7 (105.9)				
RA Treatment^∗^ Non-poor		0.08 (0.03)∗∗∗		1.21 (0.37)∗∗∗			−151.3 (64.2)∗∗				
LG Treatment^∗^ Poor		−0.01 (0.03)		−0.39 (0.59)			35.0 (77.4)				
LG Treatment^∗^ Non-poor		0.03 (0.02)∗∗		−0.47 (0.4)			7.6 (43.1)				
Poor		0.008 (0.02)		−1.93 (0.36)∗∗∗			−80.7 (69.1)				
Mean DV (Treat = 0) Controls:	0.125 No	0.125 No	32.9 No	32.9 No	4.85 No	156.7 No	159.2 No	111.0 No	−1.39 No	−1.65 No	0.001 No
Tests of Equality (p-values):											
RA^∗^ Non-Poor = RA^∗^ Poor		0.01		0.49			0.33				
LG^∗^ Non-Poor = LG^∗^ Poor		0.29		0.90			0.77				
RA^∗^ Non-Poor = LG^∗^ Non-Poor		0.15		0.002			0.03				
RA^∗^ Poor = LG^∗^ Poor		0.62		0.14			0.45				
RA^∗^ Poor = RA^∗^ Non-poor =		0.07		0.02			0.16				
LG^∗^ Poor = LG^∗^ Non-poor											
N	1858	1858	1858	1858	1858	1780	1742	1443	1886	1872	2043

Notes: We report results from OLS regressions (equations (1), (2))). The dependent variables are: *Toilet Construction* which equals 1 if the household built a toilet since baseline and 0 otherwise; *Intolerance of Open Defecation* which is the sum of responses to 9 questions about attitudes toward open defecation (45 is the maximum score possible and is the highest level of intolerance while 9 is the minimum score possible and reflects total acceptance of open defecation); *Knowledge of Causes of Diarrhea* which is a score out of 6 based on six questions about possible causes of diarrhea (a score of 6 indicates that the respondent got all of the questions correct); roundworm prevalence (eggs/g); hemoglobin (g/l); weight and height z-scores of children 0–5; and an index of the roundworm, hemoglobin, weight and height z-scores. RA (LG) treatment indicates villages are assigned to implementation by a resource agency (local government). Standard errors are clustered at the village level and are reported in parentheses. ^∗∗∗^indicates significance at 1% level, ^∗∗^ at 5% level, ^∗^ at 10% level.

**Table A2:** Social Capital Measures.

Variables	All Households			No Sanitation at Baseline		
	Mean (Treatment)	Mean (Control)	p-value Difference	Mean (Treatment)	Mean (Control)	p-value Difference
Did a household member participate in a religious group in the last 12 months?	0.77	0.76	0.74	0.71	0.76	0.28
Did a household member participate in a women's group in the last 12 months?	0.57	0.54	0.63	0.57	0.54	0.73
Did a household member participate in a rotating savings (arisan) group in the last 12 months?	0.72	0.65	0.15	0.68	0.64	0.47
How many close friends do you have in the community?	5.49	5.66	0.68	5.58	5.45	0.83
If you suddenly needed to borrow money (without interest) to meet household expenses is there someone in the community (other than family) who would be prepared to help you? (Yes = 3/Maybe = 2/No = 1)	2.50	2.54	0.58	2.51	2.51	0.95
Village Social Capital Index	0.01	−0.02	0.47	−0.03	−0.01	0.71
Observations	721	728		289	307	

Notes: This table shows the means for each of the variables which are used to generate the village social capital index, for all households as well as for the sub-sample of households that did not have private sanitation facilities at baseline. It also presents the means of the index. The p-values in columns 3 and 6 are generated from tests of statistical difference between treatment and control communities. ^∗∗∗^indicates difference is significant at 1% level, ^∗∗^ at 5% level, ^∗^ at 10% level.

**Table A3:** Balance Table – Social Capital Sample.

Variables	All Households			No Sanitation at Baseline		
	Mean (Treatment)	Mean (Control)	p-value Difference	Mean (Treatment)	Mean (Control)	p-value Difference
*Baseline Sanitation Variables:*						
Do not have own sanitation facility	0.52	0.51	0.89	1.00	1.00	.
Open defecate	0.40	0.42	0.74	1.00	1.00	.
Access to unimproved sanitation	0.11	0.14	0.37	0.00	0.00	.
Access to improved sanitation	0.49	0.43	0.27	0.00	0.00	.
Wash hands after defecation	0.99	0.99	0.52	0.97	0.99	0.31
Access to piped water	0.04	0.07	0.37	0.03	0.04	0.69
Intolerance of Open Defecation	33.3	32.6	0.27	30.3	30.0	0.64
Believes open defecation causes diarrhea	0.68	0.65	0.36	0.60	0.52	0.10^∗^
Knowledge of causes of diarrhea	4.64	4.61	0.82	4.29	4.24	0.78
*Baseline Household Characteristics:*						
Household head's age	40.23	40.12	0.89	40.11	38.45	0.13
Household head male	0.95	0.97	0.14	0.95	0.99	0.05^∗^
Household head's educational attainment:						
Elementary	0.51	0.51	0.85	0.63	0.63	0.87
Lower secondary	0.20	0.20	0.75	0.17	0.17	0.93
Upper secondary	0.17	0.20	0.47	0.11	0.10	0.91
Tertiary	0.05	0.04	0.43	0.01	0.01	0.43
Household size	4.94	4.82	0.26	4.91	4.64	0.07^∗^
Number of children in the household:						
Aged 0–5 years	1.15	1.13	0.33	1.13	1.07	0.01^∗∗∗^
Aged 6–10 years	0.35	0.30	0.13	0.37	0.28	0.05^∗^
Aged 11–17 years	0.37	0.38	0.78	0.41	0.41	0.93
Per capita household income (mill Rp/year)	2.78	2.96	0.51	1.77	1.85	0.61
House has a dirt floor	0.22	0.24	0.60	0.32	0.32	0.95
House has a tiled floor	0.05	0.06	0.49	0.03	0.04	0.82
House has walls of brick or wood	0.89	0.88	0.83	.0.80	0.82	0.61
Household uses wood as a cooking fuel	0.57	0.55	0.59	0.69	0.63	0.30
Cash transfer program (BLT) recipient	0.24	0.26	0.61	0.39	0.37	0.63
*Baseline Village Characteristics:*						
Village population	925.61	1052.51	0.21	835.33	894.69	0.46
Paved road to the nearest city	0.92	0.95	0.46	0.92	0.96	0.52
% of the village population that are muslim	97.72	97.26	0.68	98.61	98.41	0.77
River runs through village	0.83	0.86	0.62	0.92	0.93	0.86
% of households in village that open defecate	15.18	14.66	0.86	24.14	21.27	0.45
Average years of education of household heads	10.13	10.45	0.26	9.67	10.40	0.06^∗^
Village land area	63,297	33,543.05	0.27	109,804	22,601.86	0.03^∗∗^
Observations	721	728		289	307	

Notes: These are summary statistics (means) using the baseline data from the social capital sample. *Intolerance of Open Defecation* is the sum of responses to 9 questions about attitudes toward open defecation (45 is the maximum score possible and is the highest level of intolerance while 9 is the minimum score possible and reflects total acceptance of open defecation); *Knowledge of causes of diarrhea* is a score out of 6 based on six questions about possible causes of diarrhea (a score of 6 indicates that the respondent got all of the questions correct). The p-values are generated from tests of statistical difference between treatment and control communities. ^∗∗∗^indicates difference is significant at 1% level, ^∗∗^ at 5% level, ^∗^ at 10% level.

## Footnotes

1 CLTS was implemented in Indonesia, Tanzania and two locations in India. Handwashing interventions were implemented in Peru, Vietnam, and Tanzania. Results from the CLTS evaluations are reported in Patil et al. (2014) and Briceno et al. (2017), for India and Tanzania respectively. Both find increases in the ownership of improved sanitation (15 percentage point increase – from 50 to 65% – in Tanzania and 19 percentage points in India – from 22 to 41%). The Indian program is called the Total Sanitation Campaign and pairs the CLTS approach with toilet construction subsidies. An additional study, Pickering et al. (2015), conducted a clustered RCT of CLTS in Mali and finds increased toilet construction, no change in diarrhea prevalence but improvements in child growth.

2 Recently there has been a push for a greater use of randomized experiments “at scale” (Davis et al., 2017; Muralidharan and Niehaus, 2017).

3 We are aware of only a small number of studies that conduct rigorous quantitative evaluations of the scaling up process, all of which highlight the many unforeseen difficulties in scaling up projects and the need to carefully evaluate the scale up process. Bold et al. (2013) find that an educational intervention increased student test scores when implemented by an NGO in Western Kenya but failed to increase scores when replicated at scale by the government. Grossman, Humphreys and Sacramont-Lutz (2015) finds that the high take-up by marginalized populations of new low-cost technology that allows constituents to engage with their local politicians could not be replicated when scaled to comprehensively cover half the country. A more promising outcome is reported in Banerjee et al. (2017) and Banerjee et al. (2016) who build on knowledge gained through previous failed attempts to effectively scale up the “Teaching at the Right Level” program. Further, Duncan and Magnuson (2013), in a review of the impacts of pre-school programs, note that the results from programs implemented for large and representative populations are generally much smaller than those found for small-scale pilot programs.

4 Many related definitions of social capital exist which generally incorporate preferences that inform interactions amongst individuals in a pro-social manner, such as altruism, trust and reciprocity; and/or a set of underlying community networks that can be used by individuals for private or public benefit (Durlauf and Fafchamps, 2005). See also Putnam et al. (1993), Coleman (1988) and Grootaert et al. (2002).

5 In Indonesia the program is called Total Sanitation and Sanitation Marketing (TSSM) or in Indonesian, Sanitasi Total & Pemasaran Sanitasi (SToPS). It consists of a CLTS demand-side component and also a supply-side component which seeks to support the development of the local sanitation market. The supply-side component was however not well developed at the time of the evaluation (Cameron et al., 2013). For more information on CLTS see http://www.communityledtotalsanitation.org/page/clts-approach.

6 East Java's 29 rural districts were divided into three groups: Phase 1 districts received the program first, Phase 2 districts received it next, and Phase 3 districts received it last. The evaluation was conducted in Phase 2 districts. Phase 2 was chosen largely on the basis of timing. Evaluating the program in Phase 2 districts provided sufficient time for the baseline survey to be conducted prior to program implementation. Many of the start-up issues confronted in Phase 1 were sorted out by Phase 2 so the evaluation provides an impact estimate which is more representative of what could be expected from a national scaling up of the program following such large-scale piloting.

7 The districts are spread across East Java and include Probolinggo, Bonodowoso, Situbondo, Banyuwangi, Ngawi, Madiun, Jombang and Blitar. For a more in-depth description of the geographic context, see Cameron and Shah (2015). For further detail on the randomization design and process see Cameron and Shah (2010).

8 These six districts are Blitar, Bonodowoso, Jombang, Ngawi, Probolinggo, and Situbondo.

9 For the attitudinal measure the respondent is asked whether s/he agrees, strongly agrees, disagrees or strongly disagrees with: Having a toilet of our own will stop my family becoming a target of gossip; Sanitation facilities in this village improves the community as there is no longer environmental pollution; Most people that I know defecate in a toilet; It is OK to defecate in the open as our ancestors did; Having our own toilet will reduce the likelihood of family members getting diarrhea; It is OK to defecate in the river as others do it; It is acceptable for children to defecate in the open; It is acceptable to defecate in the open if you don't have a toilet; People who defecate in the open will not be accepted by the community. The aggregated attitudinal measure is the sum of responses to these 9 questions about attitudes toward open defecation (oriented so a higher score indicates greater intolerance of open defecation). 45 is the maximum score possible and is the highest level of intolerance while 9 is the minimum score possible and reflects total acceptance of open defecation. For the knowledge of the causes of diarrhea the caregiver is asked to indicate whether the following activities cause diarrhea: drinking dirty water; using dirty latrines; other people defecating in the river; other people defecating in another open space (yard/rice field/beach/etc); not washing hands with water; not washing hands with soap. The aggregated knowledge measure is a score out of 6 with a score of 6 indicating that the respondent got all of the questions correct.

10 Owing to CLTS forming part of a Gates Foundation global learning agenda, the CLTS program itself and the scale up strategy are unusually well-documented. For example, see Kar and Chambers (2008); Rosenzweig and Kopitopoulos (2010); Mukherjee (2009, 2011); Pinto (2013); for a global discussion see Chambers (2009). The WHO scale-up strategy includes identifying, documenting and assessing the nature of the innovation to be scaled up; increasing the capacity of the implementing agency; assessing the broader environment in which the project is to be scaled up; supporting the resource team which will support the scale up; embedding the project within the institutions of the target country; and documenting the scale up strategy.

11 The categorization here draws from and augments Grossman et al. (2015) who attribute the lower uptake in the scaled up version of the political engagement technology they study to a design effect—invitations to participate were given over the radio, rather than in person during a survey and an implementation agent effect— scale up involved implementation by parliament and promotion by politicians which may have altered incentives. The lack of replicability found in the teaching intervention in Bold et al. (2013) is slated to a combination of general equilibrium effects arising from political economy forces associated with union resistance to the hiring of a large number of contract teachers, and implementation agent effects. A further related study, Berge et al. (2012), examines implementation agency effects but in the context of a small-scale business training program in Tanzania. It finds that the training was much more effective when implemented by professional trainers rather than by a local NGO.

12 Seehttp://millionssaved.cgdev.org/case-studies/indonesias-total-sanitation-and-sanitation-marketing-program.

13 Note that most households in our sample are poor in the sense of being below the national poverty line. Here we are defining “poor” to capture the poorer households within our sample.

14 The estimated poor performance of LGs relative to RAs in terms of toilet construction, attitudes towards open defecation and roundworm prevalence could reflect lesser adherence by the local governments to the treatment assignment. To investigate this possibility we estimated two stage least squares regressions where we instrument for whether a triggering (by either a RA or LG) was confirmed by community survey respondents as having taken place with whether the village was assigned to be a treatment village, and its interaction with whether the village was assigned to be triggered by a RA. This strategy allows for the differential effect on the probability of a treatment village actually being triggered depending on whether it was to be triggered by a resource agency or local government. The instruments are strongly predictive and the second stage results are consistent with the OLS results. Results are available upon request.

15 Isham and Kähkönen (1999) and Isham and Kähkönen (2002) use cross-sectional data from villages participating in community-based water interventions in Indonesia, India, and Sri Lanka, respectively, and find that higher levels of social capital are positively associated with greater household participation in the selection of the type of water infrastructure and construction monitoring, and that this can lead to greater health improvements. Pargal, Huq and Gilligan (1999) find that social capital is positively associated with voluntary solid waste management systems arising in Dhaka, Bangladesh. Evidence from case studies is mixed (Uphoff and Wijayaratna, 2000).

16 In addition to seeking to improve living standards in poor communities, participatory development projects are often explicitly viewed as a vehicle for building social capital. By empowering communities and providing a reason and process by which community members can work together for a common goal, participatory development provides a potential mechanism for increasing community member interactions, forging relationships and building trust (Avdeenko and Gilligan, 2015). In the long-run, gains in social capital might facilitate economic development and help to sustain program impacts (Dongier et al., 2003; Mansuri and Rao, 2004)). In results reported in a working paper now subsumed by this paper (Cameron et al., 2015) we explore this issue in the context of CLTS and find that the program did not build social capital. In fact, treatment reduced trust in already low social capital settings. For surveys of evidence on this issue see Wong (2012), Mansuri and Rao (2004), and Mansuri and Rao (2012).

17 The method of Kling et al. (2007) is used to construct the indices in Table 5.

18 We also directly examine the construction of public and shared toilets. More active communities do not build more public and shared toilets.

19 For discussion of data collection efforts to identify implementation and scale up difficulties see Bamberger et al. (2010); Pritchett et al. (2013); Andrews et al. (2012); Woolcock (2013); Kruk et al. (2016); Brown et al. (2014). Banerjee et al. (2016) used process data to identify the keys to scale up success.
